# Supplementary material for: Light-steered locomotion of muscle-like hydrogel by self-coordinated shape change and friction modulation
Source: Nat Commun. 2020 Oct 14;11:5166. doi: 10.1038/s41467-020-18801-1 (PMC7560679; doi:10.1038/s41467-020-18801-1)
Supplement: Supplementary file 1 — Supplementary Information [file 41467_2020_18801_MOESM1_ESM.pdf]

Supplementary Information

Light-steered locomotion of muscle-like hydrogel by self-coordinated shape change and friction modulation

Zhu *et al.*

Supplementary Figures

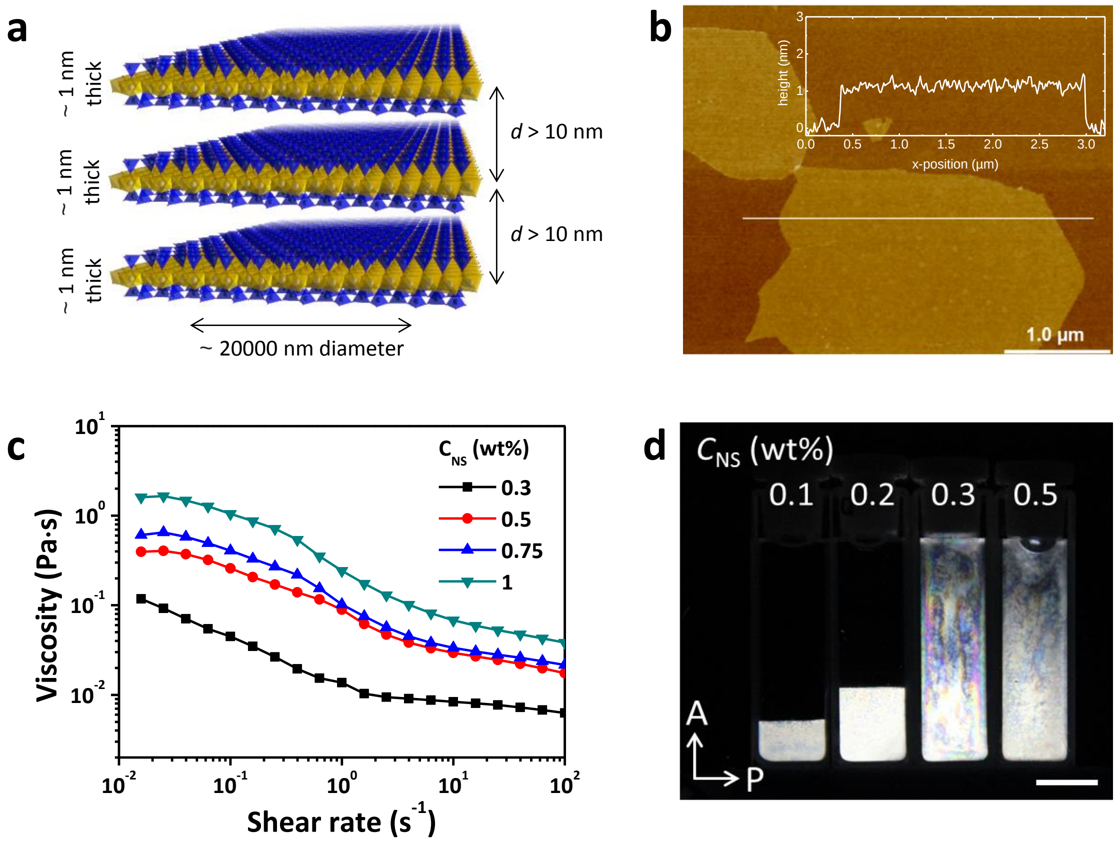

Supplementary Fig. 1. Structure and properties of fluorohectorite $[Na_{0.5}][Li_{0.5}Mg_{2.5}][Si_4]O_{10}F_2$ nanosheets and its aqueous suspensions. a, Schematic structure of negatively charged nanosheets. **b**, Topographical atomic force microscope (AFM) image of the nanosheet showing the thickness and lateral dimensions. **c**, Viscosity of the aqueous suspensions with different content of nanosheet, C_{NS}, as a function of shear rate. **d**, Image of the suspensions with different C_{NS} after storage for one week observed between crossed polarizer and analyzer. A: analyzer; P: polarizer. Scale bars in (b) and (d) are 1 μm and 1 cm, respectively.

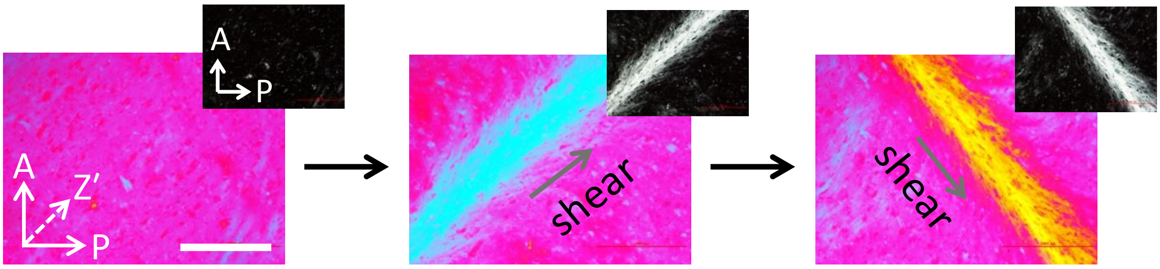

Supplementary Fig. 2. Mechanical shear-induced localized orientation of the nanosheet suspension. Aqueous suspension with C_{NS} of 0.3 wt% is dropped on a glass substrate, and a mechanical shear is applied to the solution film by a syringe needle, which results in localized orientation of NSs along the shearing direction and strong birefringence under POM. A: analyzer; P: polarizer; Z': slow axis of the 530 nm tint plate. Scale bar, 1 mm.

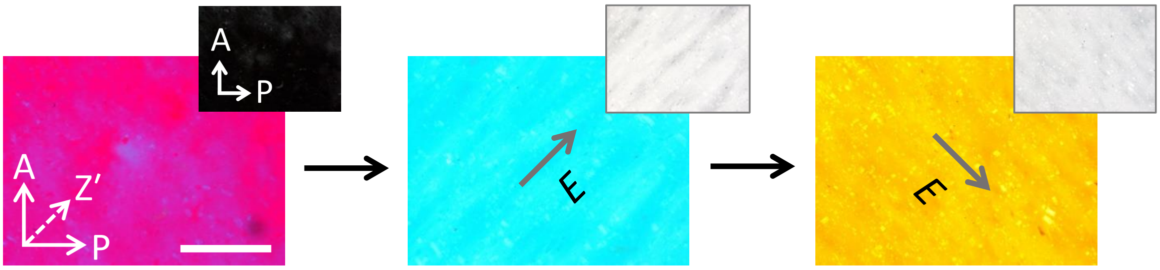

Supplementary Fig. 3. Electric-field-induced orientation of the nanosheet suspension. Aqueous suspension with C_{NS} of 0.3 wt% is injected into a cell consisting of a pair of parallel glasses with 2 mm silicon spacer. Then a high-frequency AC electric field is applied to the suspension to orient the NSs. Reorientation of the NSs is achieved by changing the electric field direction applied to the suspension. The distance between the Ag electrodes is 20 mm, the voltage amplitude of the electric field is 50 V, the frequency of the electric field is 10 kHz, and the field action time is 60 min. Scale bar, 1 mm.

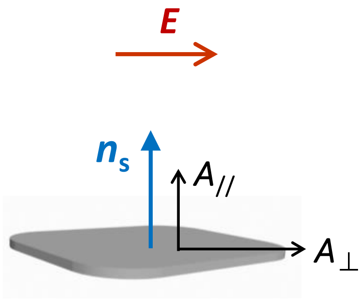

Supplementary Fig. 4. Illustration of the orientation of nanosheet under the electric field. The orientation of highly charged colloidal particles under a high-frequency AC electric field can be explained by Maxwell-Wagner-O'Konski polarization mechanism. The mobile counterions around the charged particle results in a strongly enhanced conductivity at the interface and also the polarization of the counterion cloud around the charged particle. Owing to the anisotropic structure of nanosheet, the excess of polarizability of particle compared to the surrounding medium is also anisotropic at different directions. The excess of polarizability of the negatively charged nanosheet, $\Delta A = A_{//} - A_{\perp}$, is negative, which dominates the orientation of colloidal particle under the electric field. The normal direction $\mathbf{n}_s$ of the NS aligns perpendicular to the electric field **E**.

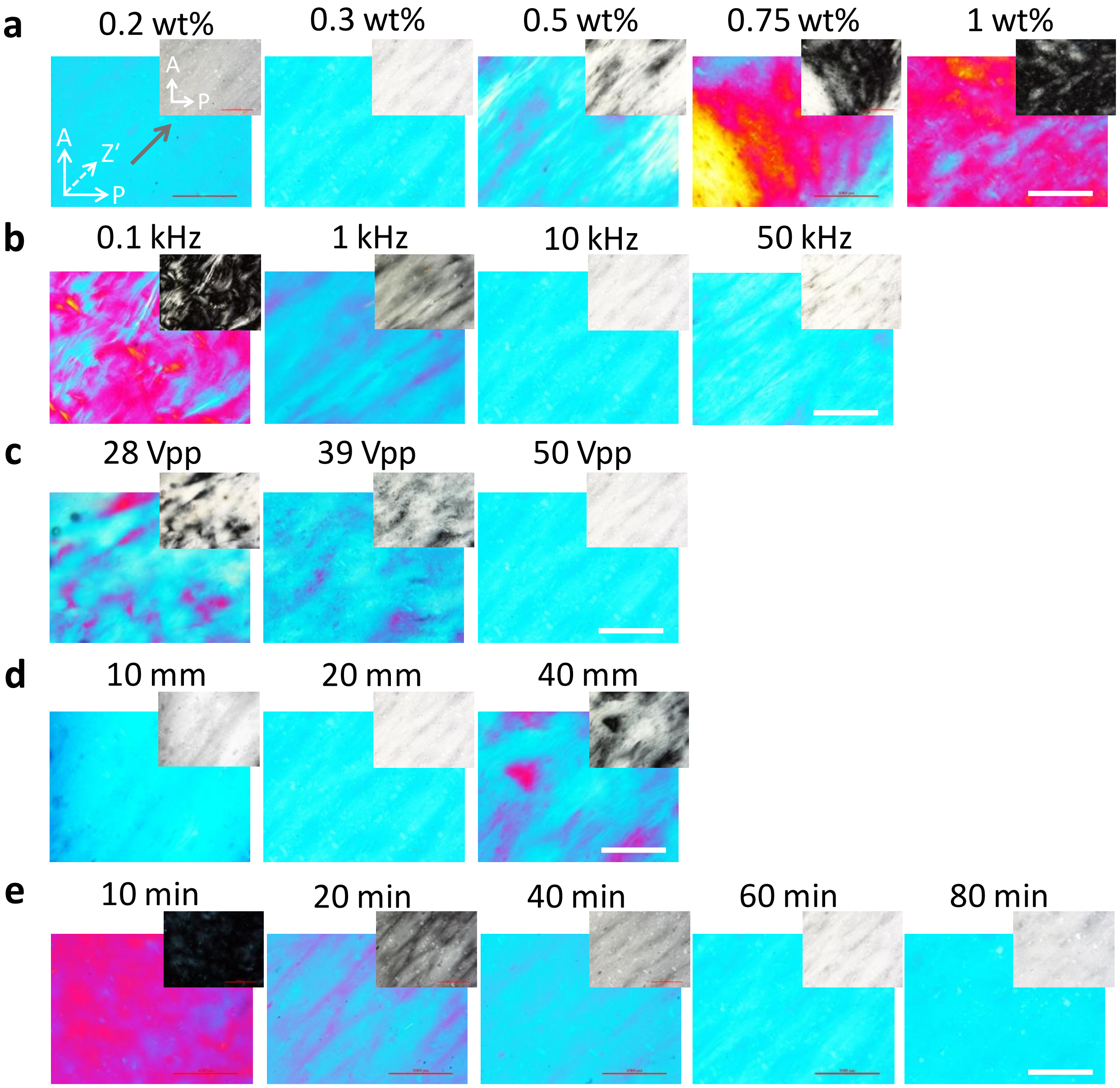

Supplementary Fig. 5. Electric-field-induced orientation of nanosheets at different experimental conditions. The experimental conditions are optimized by examining the birefringence of the nanosheet suspension with varying NS content C_{NS} (**a**) under the AC electric field with different frequency (**b**), voltage amplitude (**c**), action time (**d**) of the AC electric field, and distance between the electrodes (**e**). The experiments are designed by changing one parameter each time with referenced condition of C_{NS} of 0.3 wt%, frequency of 10 kHz, voltage amplitude of 50 V, action time of 60 min, and electrode distance of 20 mm. Scale bar, 1 mm.

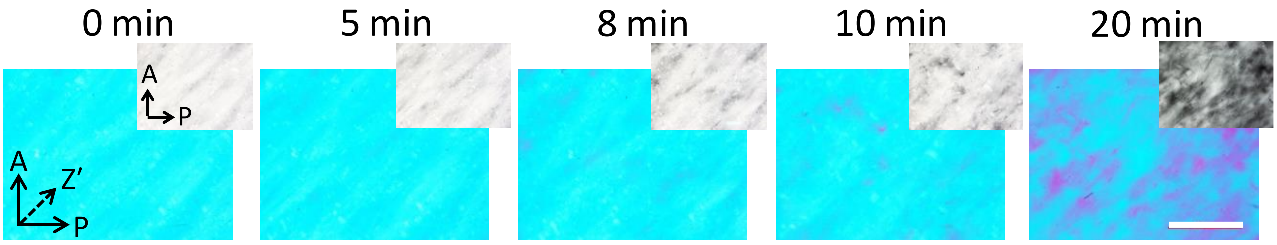

Supplementary Fig. 6. Structural relaxation of the electrically oriented nanosheets. The NSs in the aqueous suspension are oriented under an AC electric field for 60 min, and the structural relaxation is monitored after the ceasing of the electric field. The relaxation time is noted above the POM images. Experimental condition: C_{NS} of 0.3 wt%, frequency of 10 kHz, voltage amplitude of 50 V, and electrode distance of 20 mm. Scale bar, 1 mm.

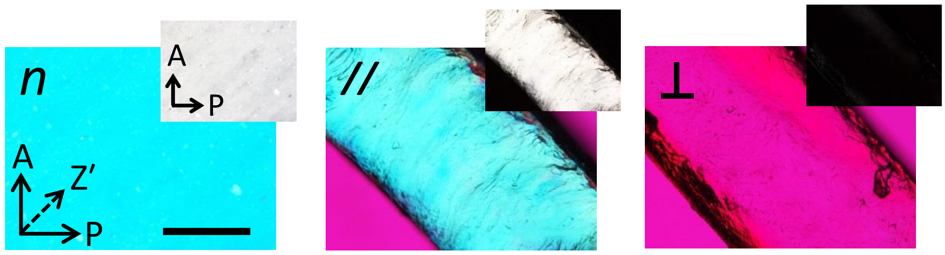

Supplementary Fig. 7. Anisotropic structure of the monodomain hydrogel in the as-prepared state. POM images of the as-prepared hydrogel viewed from different directions. Scale bar, 1 mm.

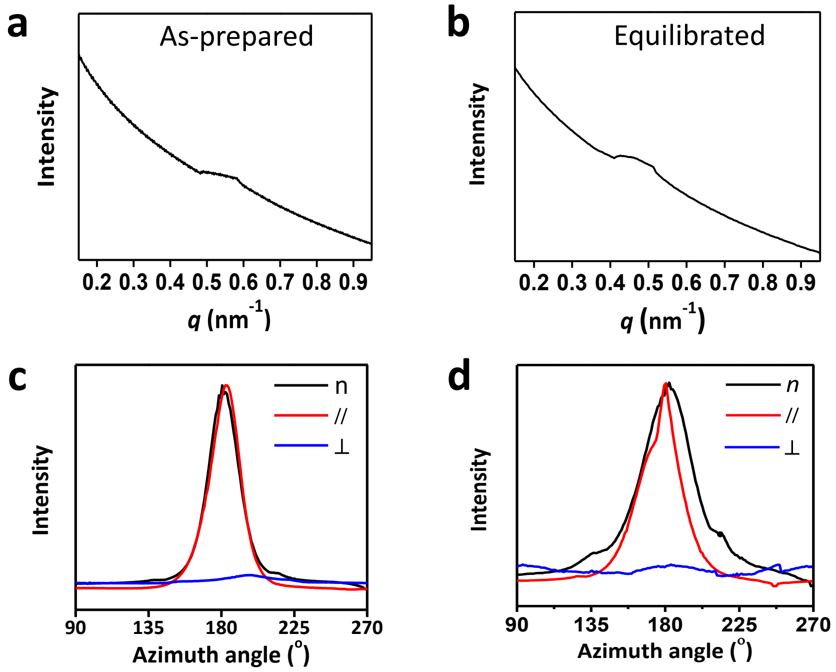

Supplementary Fig. 8. Small angle X-ray scattering intensity profiles of the monodomain hydrogel. a,**b**, Intensity profiles of the monodomain hydrogel in the as-prepared state (a) and equilibrated state (b). **c**,**d**, Scattering intensity-azimuth angle plots of the monodomain hydrogel in the as-prepared state (c) and equilibrated state (d).

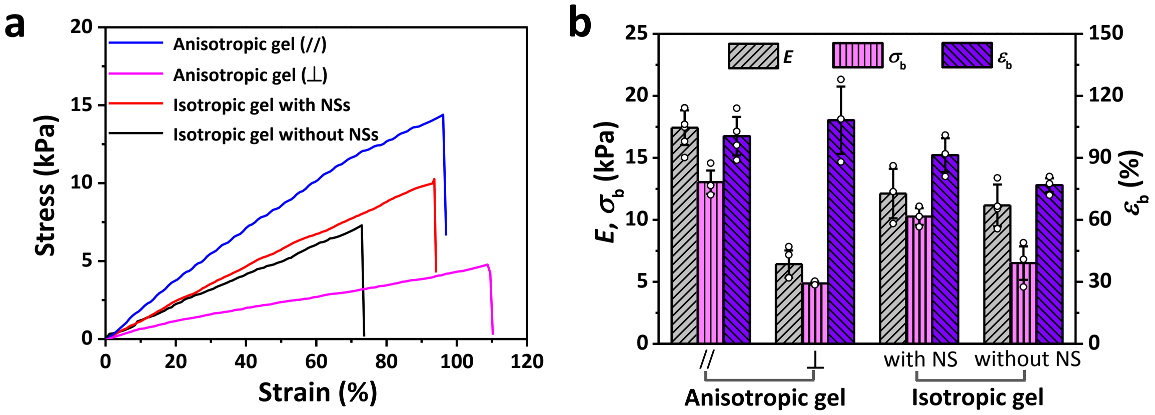

Supplementary Fig. 9. Anisotropic mechanical properties of the monodomain hydrogels. a, Tensile stress-strain curves of the monodomain hydrogel stretched from the direction parallel (//) and perpendicular (⊥) to the alignment of NS. The stress-strain curves of isotropic PNIPAAm hydrogels with and without NS are also presented for comparison. **b**, Mechanical parameters, including the Young's modulus *E*, the breaking stress σ_b, and the breaking strain ε_b, of the hydrogels. The tensile tests are performed to the gel samples at room temperature with a stretch rate of 100 mm min^{-1}. The samples have a gauge length of 12 mm and a width of 2 mm. Error bars represent the standard deviation of the mean.

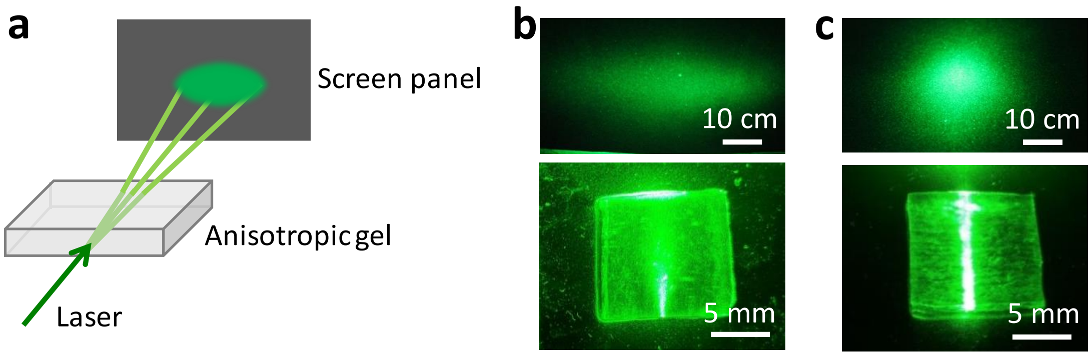

Supplementary Fig. 10. Anisotropic optical property of the monodomain hydrogel. a, Schematic for the experiment. A rectangular anisotropic hydrogel (dimensions: 10 mm × 10 mm × 2 mm) is irradiated by a 520 nm green-light laser from the lateral direction, parallel (//) or perpendicular (⊥) to the alignment of NSs. The light path through the hydrogel is observed from the top of the gel sheet; the scattering pattern is displaced on a screen panel normal to the laser beam with a sample-to-panel distance of 15 cm. **b**,**c**, Photos of the light path through the gel (the bottom) and the scattering pattern on the screen panel (the top), when the light is irradiated from the // direction (b) and the ⊥ direction (c). When the gel is irradiated by a laser from the ⊥ direction, a clear light path is observed from the top (*n* direction), accompanying with a bright circular light spot on the screen panel perpendicular to the laser. By contrast, when the gel is irradiated from the // direction, the light path is hazy, accompanying with vague elliptical light spot on the screen panel. This anisotropic light scattering arises from the highly ordered structure of NSs within the gel.

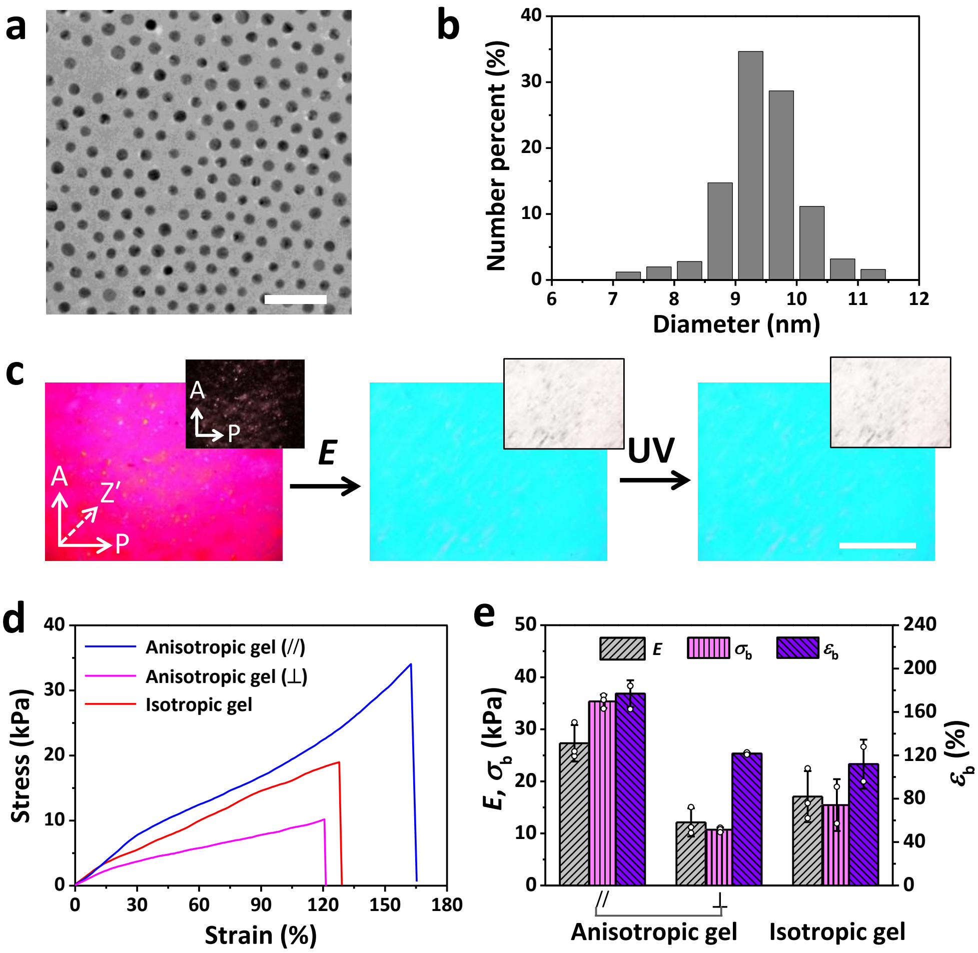

Supplementary Fig. 11. Synthesis and mechanical properties of the photoresponsive monodomain hydrogel. a,**b**, TEM image (a) and size distribution (b) of the gold nanoparticles (AuNPs). The diameter of AuNPs is 9.5 ± 0.7 nm (average ± standard deviation; n = 485). **c**, POM images to show the electric-field-induced orientation of NSs and synthesis of monodomain hydrogel by polymerization of the precursor solution with AuNPs. **d**,**e**, Tensile stress-strain curves (d) and corresponding mechanical parameters (e) of the AuNP-containing PNIPAAm hydrogels with anisotropic and isotropic NSs. Scale bars in (a) and (c) are 50 nm and 1 mm, respectively. Error bars represent the standard deviation of the mean.

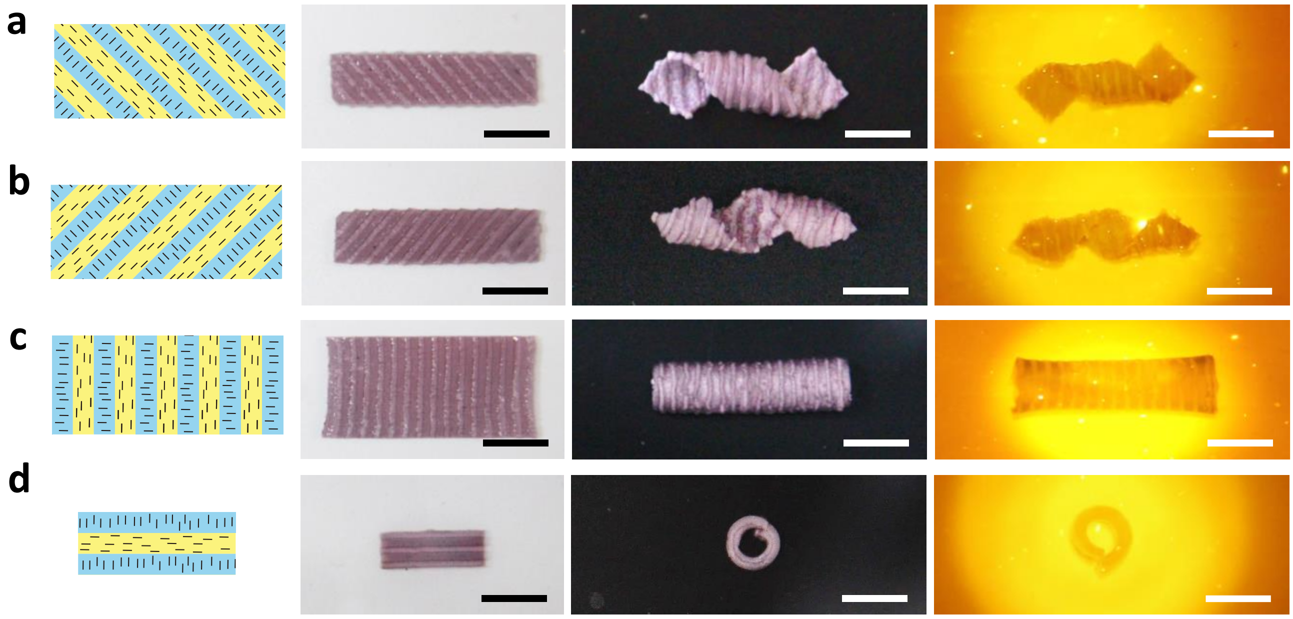

Supplementary Fig. 12. Programmed deformation of stripe-patterned hydrogels into 3D configurations. The patterned hydrogels have orthogonal alignments of NSs parallel or perpendicular to the stripe direction. The stripes align with an angle, θ, of –45° (a), 45° (b), 0° (c), or 90° (d) relative to the long axis of the slender patterned hydrogel. After being incubated in 40 ^{o}C water bath or irradiated by 520 nm green light, the patterned hydrogels deform into right-handed helix, left-handed helix, long tube, and short roll, respectively. The first column shows the alignments of NSs in the patterned hydrogels; the second, third, and forth columns are the photos of the hydrogel before the actuation, in hot water, and under light irradiation, respectively. Scale bar, 1 cm.

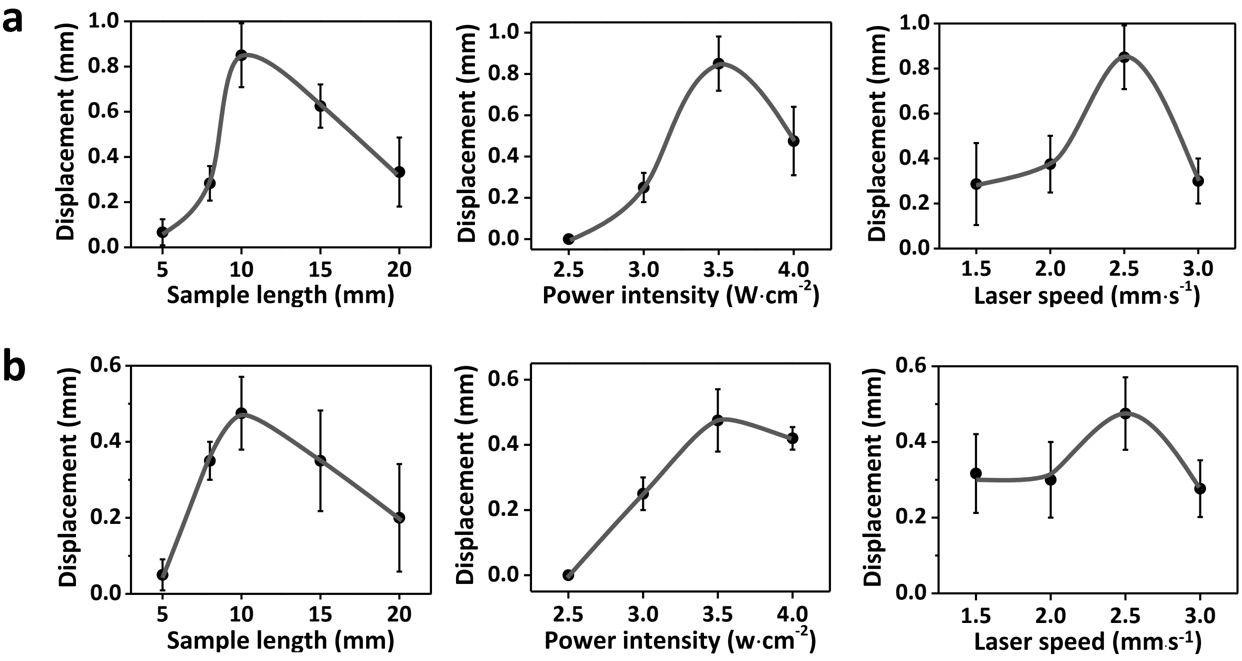

Supplementary Fig. 13. Effects of sample length, power intensity, and scanning speed on the crawling motions of the monodomain hydrogels. a, Displacement of hydrogel with NS aligned along the long axis of the slender gel sheet over one cycle of light scanning. **b**, Displacement of hydrogel with NS aligned perpendicular to the long axis of the slender gel sheet per cycle of light scanning. The referenced experimental conditions: sample dimensions, 10 mm × 5 mm × 1 mm; power intensity, 3.5 W cm^{-2}, scan speed, 2.5 mm s^{-1}. Error bars represent the standard deviation of the mean.

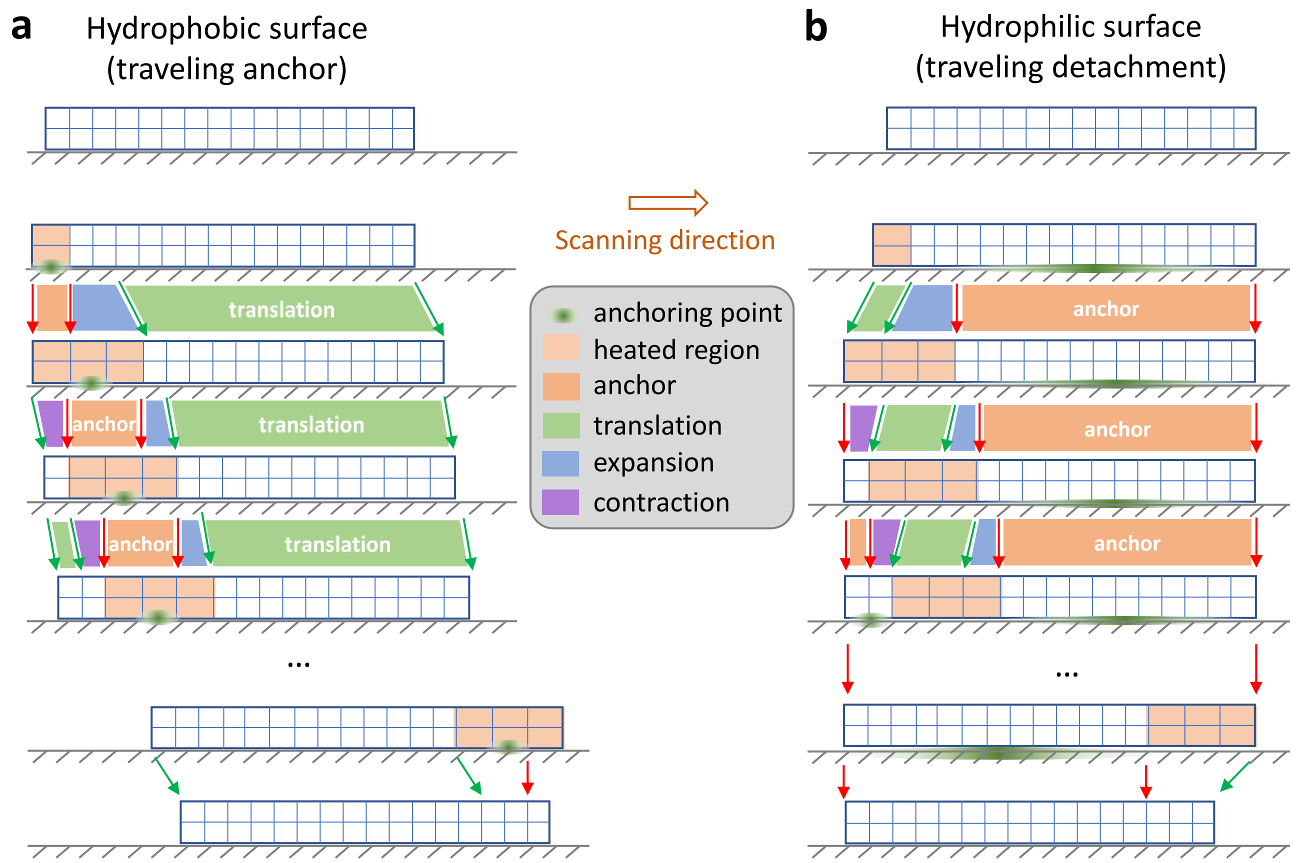

Supplementary Fig. 14. Schematics of the crawling mechanism of a monodomain hydrogel. a, Forward crawling of a gel on a hydrophobic surface, on which the heated region exhibits higher friction and acts as a traveling anchor. **b**, Backward crawling of a gel on a hydrophilic surface, on which the heated region of low friction acts as a traveling area of detachment. The scanning direction of the light beam is from left to right, and the NSs in the monodomain gel align in the transverse direction in both cases. The crawling distance in each cycle scales with the strain times the total length in the traveling-anchor mechanism, much more effective than that induced by the traveling-detachment mechanism, which scales with the strain times the size of the heated region.

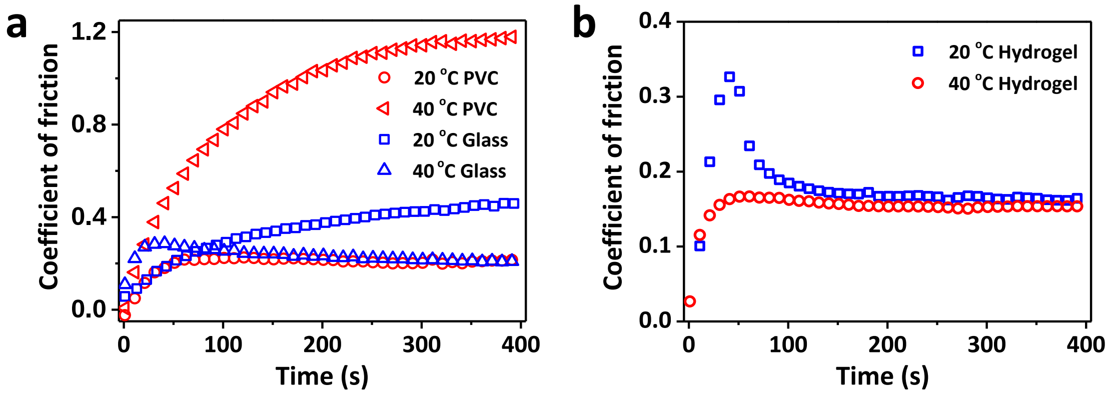

Supplementary Fig. 15. Friction coefficient of PNIPAAm hydrogel containing randomly dispersed NSs and AuNPs against different substrates. a, Variations of the friction coefficient of the hydrogel against a hydrophobic PVC substrate and hydrophilic glass substrate as a function of test time at 20 and 40 ^{o}C. **b**, Variations of the friction coefficient of the hydrogel against a hydrophilic double-network hydrogel sheet as a function of test time at 20 and 40 ^{o}C.

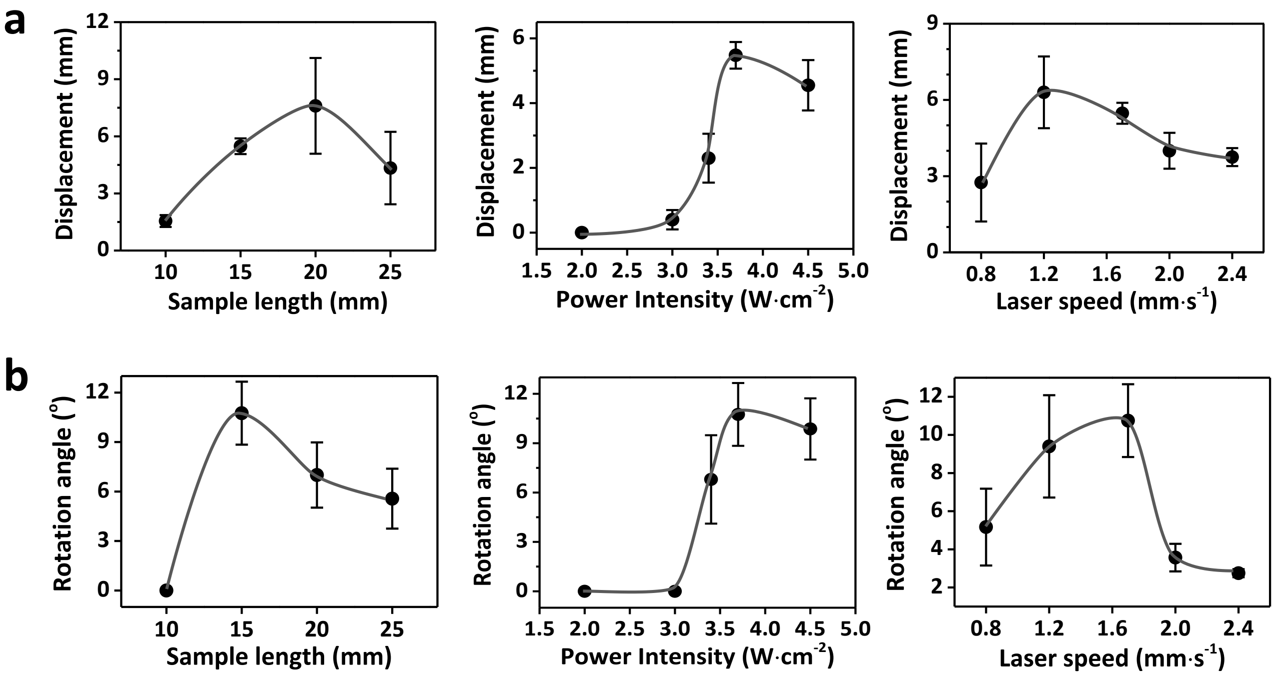

Supplementary Fig. 16. Effects of sample length, power intensity, and scanning speed on the walking and rotating motions of the patterned anisotropic hydrogel. a, Walking stride of the hydrogel over one cycle of light scanning. **b**, Rotation amplitude of the hydrogel over one cycle of light scanning. The referenced experimental conditions: sample dimensions, 10 mm × 5 mm × 1 mm; power intensity, 3.5 W cm^{-2}, scan speed, 1.7 mm s^{-1}. Error bars represent the standard deviation of the mean.

Supplementary Tables

Supplementary Table 1. Comparison of the locomotion speed of gel-based soft robots in this work and other ones in the literatures

System	Locomotion mode	Stimulation	locomotion speed	Kinematics	Ref.
P(NIPAAm-*co*-Ru(bpy)$_3$-*co*-AMPS) gel	walking	Belousov-Zhabotinsky reaction	2.8×10^{-3} mm s^{-1} (4.7×10^{-4} BL s^{-1})[a]	ratchet substrate	1
Liquid crystal gel	walking	light	0.3 mm s^{-1} (1.4×10^{-2} BL s^{-1})	ratchet substrate	2
Spiropyran-based PNIPAAm gel	walking	light	3.3×10^{-4} mm s^{-1} (2.1×10^{-4} BL s^{-1})	ratchet substrate	3
	turning	light	4.6×10^{-4} rad s^{-1}		
Spiropyran-based P(NIPAAm-*co*-acrylic acid) gel	walking	light	2.5×10^{-4} mm s^{-1} (8.9×10^{-5} BL s^{-1})	ratchet substrate	4
Hybrid polyelectrolyte gel	walking	electric field	0.04 mm s^{-1} (4.1×10^{-3} BL s^{-1})	asymmetric shape	5
Polyelectrolyte gel	walking	electric field	0.13 mm s^{-1} (1.3×10^{-2} BL s^{-1})	ratchet substrate	6
Polyelectrolyte gel	walking	electric field	0.42 mm s^{-1} (2.1×10^{-2} BL s^{-1})	ratchet substrate	7
Monodomain nanocomposite PNIPAAm gel	walking	temperature	3.7×10^{-2} mm s^{-1} (2.7×10^{-4} BL s^{-1})	asymmetric shape	8
PNIPAAm gel	crawling	temperature	1.9×10^{-3} mm s^{-1} (5.6×10^{-4} BL s^{-1})	geometric confinement	9
PNIPAAm gel	crawling	temperature	6.7×10^{-3} mm s^{-1} (2.2×10^{-5} BL s^{-1})	geometric confinement	10
Monodomain nanocomposite PNIPAAm gel	crawling	light	0.28 mm s^{-1} (1.9×10^{-2} BL s^{-1})	geometric confinement	11
Anisotropic nanocomposite PNIPAAm gel	crawling	light	6.2×10^{-2} mm s^{-1} (6.2×10^{-3} BL s^{-1})	self-coordinated friction manipulation	this work
	walking		0.37 mm s^{-1} (2.5×10^{-2} BL s^{-1})		
	turning		9.4×10^{-3} rad s^{-1}		

[a] BL s^{-1} means body length per second, as calculated by the linear speed divided by the body length of the soft robot.

Supplementary Table 2. Recipe of precursor solutions for the synthesis of gels

Components	PNIPAAm gel	NS gel	NS/AuNP gel
NIPAAm	1.1318 g	1.1318 g	1.1318 g
MBAA	0.0462 g	0.0462 g	0.0462 g
LAP	0.0147 g	0.0147 g	0.0147 g
NS	–	0.3 wt%	0.3 wt% [a]
AuNP	–	–	0.013 wt% [a]
water	10 g	10 g	10 g

[a] The wt% represents the content of NS or AuNP in 10 g water.

Supplementary References

1. Maeda, S., Hara, Y., Sakai, T., Yoshida, R. & Hashimoto, S. Self-walking gel. *Adv. Mater.* **19**, 3480–3484 (2007).
2. Shahsavan, H. *et al.* Bioinspired underwater locomotion of light-driven liquid crystal gels. *Proc. Natl. Acad. Sci. U.S.A.* **117**, 5125-5133 (2020).
3. Li, C. *et al.* Supramolecular-covalent hybrid polymers for light-activated mechanical actuation. *Nat. Mater.* **19**, 900–909 (2020)
4. Wayne, F. *et al.* Spiropyran based hydrogels actuators–Walking in the light. *Sensor. Actuat. B-Chem.* **250**, 608–616 (2017).
5. Morales, D., Palleau, E., Dickey, M. D. & Velev, O. D. Electro-actuated hydrogel walkers with dual responsive legs. *Soft Matter* **10**, 1337–1348 (2014).
6. Yang, C. *et al.* Hydrogel walkers with electro-driven motility for cargo transport. *Sci. Rep.* **5**, 13622 (2015).
7. Osada, Y., Okuzaki, H. & Hori, H. A polymer gel with electrically driven motility. *Nature* **355**, 242–244 (1992).
8. Kim, Y. S. *et al.* Thermoresponsive actuation enabled by permittivity switching in an electrostatically anisotropic hydrogel. *Nat. Mater.* **14**, 1002–1007 (2015).
9. Arora, H. *et al.* Earthworm inspired locomotive motion from fast swelling hybrid hydrogels. *J. Polym. Sci., Part A: Polym. Chem.* **47**, 5027–5033 (2010).
10. Vernerey, F. & Shen, T. The mechanics of hydrogel crawlers in confined environment. *J. R. Soc. Interface* **14**, 20170242 (2017).
11. Sun, Z. *et al.* An anisotropic hydrogel actuator enabling earthworm-like directed peristaltic crawling. *Angew. Chem. Int. Ed.* **130**, 15998–16002 (2018).
