# Supplementary material for: Light-steered locomotion of muscle-like hydrogel by self-coordinated shape change and friction modulation
Source: Nat Commun. 2020 Oct 14;11:5166. doi: 10.1038/s41467-020-18801-1 (PMC7560679; doi:10.1038/s41467-020-18801-1)
Supplement: Supplementary file 3 — Description of Additional Supplementary Files [file 41467_2020_18801_MOESM3_ESM.pdf]

Description of Additional Supplementary Files

File Name: Supplementary Movie 1
Description: **Reversible anisotropic deformation of the AuNP-containing monodomain hydrogel under cyclic light irradiation.** The hydrogel is incubated in a 25 ^{o}C water bath for the light-induced deformation. A cut-off filter is equipped on the camera to filter out the strong green light for better visualization of the hydrogel under light irradiation. Gel dimensions: 5 mm × 5 mm × 2 mm; light wavelength, 520 nm; light intensity, 3.7 W cm^{-2}; movie speed, 5×.

File Name: Supplementary Movie 2
Description: **Forward crawling of the slender monodomain hydrogel on a PVC substrate under cyclic scanning of a light beam.** The monodomain hydrogel sheet with longitudinally aligned NSs is placed on a hydrophobic PVC substrate and incubated in a 25 ^{o}C water bath for the light-steered crawling motion. Gel dimensions, 10 mm × 5 mm × 1 mm; power intensity, 3.5 W cm^{-2}; scanning speed, 1.7 mm s^{-1}; movie speed, 5×.

File Name: Supplementary Movie 3
Description: **Retrograde crawling of the slender monodomain hydrogel on a PVC substrate under cyclic scanning of a light beam.** The monodomain hydrogel sheet with laterally aligned NSs is placed on a hydrophobic PVC substrate and incubated in a 25 ^{o}C water bath for the light-steered crawling motion. Gel dimensions, 10 mm × 5 mm × 1 mm; power intensity, 3.5 W cm^{-2}; scanning speed, 1.7 mm s^{-1}; movie speed, 5×.

File Name: Supplementary Movie 4
Description: **Numerical simulation showing the forward crawling of the slender monodomain hydrogel on a hydrophobic substrate under the scanning of a light beam.** The hydrogel with longitudinally aligned NSs shows traveling extension deformation and anchoring point under the scanning of light. The colors indicate the normalized temperature.

File Name: Supplementary Movie 5
Description: **Numerical simulation showing the retrograde crawling of the slender monodomain hydrogel on a hydrophobic substrate under the scanning of a light beam.** The hydrogel with laterally aligned NSs shows traveling contraction deformation and anchoring point under the scanning of light. The colors indicate the normalized temperature.

File Name: Supplementary Movie 6
Description: **Traveling deformation yet no displacement of the slender monodomain hydrogel on a tough hydrogel under cyclic scanning of a light beam.** The monodomain hydrogel with laterally aligned NSs is placed on a hydrophilic, tough hydrogel and incubated in a 25 ^{o}C water bath for the light-steered deformation. Gel dimensions, 10 mm × 5 mm × 1 mm; power intensity, 3.5 W cm^{-2} ; scanning speed, 1.7 mm s^{-1}; movie speed, 5×.

File Name: Supplementary Movie 7
Description: **Forward crawling of the slender monodomain hydrogel on a glass substrate under cyclic scanning of a light beam.** The hydrogel with laterally aligned NSs is placed on a hydrophilic glass substrate and incubated in a 25 ^{o}C water bath for the light-steered crawling motion. Gel dimensions, 10 mm × 5 mm × 1 mm; power intensity, 3.5 W cm^{-2}; scanning speed, 1.7 mm s^{-1}; movie speed, 5×.

File Name: Supplementary Movie 8
Description: **Light-steered walking of the stripe-patterned hydrogel sheet on a PVC substrate under the cyclic scanning of a light beam**. The patterned hydrogel is placed on a hydrophobic PVC substrate and incubated in a 25 ^{o}C water bath for the light-steered walking motion with controlled direction. Gel dimensions, 15 mm × 5 mm × 1 mm; power intensity, 3.5 W cm^{-2}; scanning speed, 1.7 mm s^{-1} ; movie speed, 5×.

File Name: Supplementary Movie 9
Description: **Numerical simulation showing the walking of the stripe-patterned hydrogel sheet on a hydrophobic substrate under the scanning of a light beam.** The hydrogel shows traveling bending deformation under the scanning of light. The colors indicate the normalized temperature.

File Name: Supplementary Movie 10
Description: **Light-steered turning motion of the stripe-patterned hydrogel sheet on a PVC substrate under the scanning of a light beam.** The patterned hydrogel is incubated in a 25 ^{o}C water bath and placed on a hydrophobic PVC substrate for the light-steered turning motion. The light is scanned along the diagonal of the patterned hydrogel sheet. Gel dimensions, 15 mm × 5 mm × 1 mm; power intensity, 3.5 W cm^{-2}; scanning speed, 1.7 mm s^{-1}; movie speed, 2×.

File Name: Supplementary Movie 11
Description: **Light-steered turning motion of the stripe-patterned hydrogel sheet on a PVC substrate under cyclic scanning of a light beam.** The patterned hydrogel is incubated in a 25 ^{o}C water bath and placed on a hydrophobic PVC substrate for the light-steered turning motion. The light is repeatedly scanned along the diagonal of the patterned hydrogel sheet. Gel dimensions, 15 mm × 5 mm × 1 mm; power intensity, 3.5 W cm^{-2}; scanning speed, 1.7 mm s^{-1}; movie speed, 5×.

File Name: Supplementary Movie 12
Description: **Numerical simulation showing the turning of the stripe-patterned hydrogel sheet on a hydrophobic substrate under the scanning of a light beam.** The coordinated sequence of localized deformation and dynamic friction of the gel results in the turning motion. The colors indicate the normalized temperature.
